# Design and Investigation of Modern UWB-MIMO Antenna with Optimized Isolation

**DOI:** 10.3390/mi11040432

**Published:** 2020-04-20

**Authors:** Muhammad Irshad Khan, Muhammad Irfan Khattak, Saeed Ur Rahman, Abdul Baseer Qazi, Ahmad Abdeltawab Telba, Abdelrazik Sebak

**Affiliations:** 1 Department of Electrical Engineering, University of Engineering and Technology, Peshawar 25000, Pakistan; M.I.Khattak@uetpeshawar.edu.pk; 2 College of Electronic and Information Engineering, Nanjing University of Aeronautics and Astronautics (NUAA), Nanjing 210016, China; saeed@nuaa.edu.cn; 3 Department of Software Engineering, Bahria University, Islamabad 44000, Pakistan; abq.buic@bahria.edu.pk; 4 Department of Electrical Engineering, King Saud University, Riyadh 11421, Saudi Arabia; atelba@ksu.edu.sa; 5 Electrical and Computer Engineering, Concordia University, Montreal, QC H3G 1M8, Canada; abdo@ece.concordia.ca

**Keywords:** Ultrawide band, super wide band, multiple input multiple output, voltage standing wave ratio, S_11_, impedance bandwidth, envelope correlation coefficient, gain

## Abstract

This paper proposes a compact, semi-circular shaped multiple input multiple output (MIMO) antenna design with high isolation and enhanced bandwidth for ultrawide band (UWB) applications. A decoupling stub is used for high isolation reaching up to −55 dB over the entire bandwidth. The proposed antenna is used for UWB as well as super wide band (SWB) applications. The overall size of the proposed antenna is 18×36×1.6 mm^3^. The $\left|S_{11}\right|\;$and voltage standing wave ratio (VSWR) of the proposed antenna are less than −10 dB and 2, respectively, in the range of 3–40 GHz. The total impedance bandwidth of the proposed design is 37 GHz. The VSWR, $\left|S_{11}\right|$, $\left|S_{22}\right|$, $\left|S_{21}\right|$, $\left|S_{12}\right|$, gain, envelope correlation coefficient (ECC), radiation pattern, and various other characteristic parameters are discussed in detail. The proposed antenna is optimized and simulated in a computer simulation technology (CST) studio, and printed on a FR4 substrate.

## 1. Introduction

Over the past few decades, the field of antennas for wireless communication systems has been transitioning towards having a single radiator for multiple applications, particularly when the need for higher speeds is in demand by consumers [1]. UWB and super wide band (SWB) antennas are examples of such antennas, and have been research considerations for the reasons stated above [2,3,4,5,6,7]. These antennas have various advantages, such as fabrication simplicity, low power consumptions, high transmission rate, and low cost [8]. Despite these benefits, SWB antennas also have some limitations, such as multi path fading and channel capacity, which affect the overall performance of such systems [9]. Recently, MIMO structures with multiple radiating elements have been introduced to combat such problems without using more bandwidth and transmitting power [10]. Commonly, MIMO antennas are used on both sides of communication systems (i.e., transmitter and receiver sides), to enhance communication quality and capacity of a channel [11]. Due to deployment of multiple radiating elements in close proximity, MIMO antenna systems adopt mutual coupling, and one of the solutions is to place radiating elements at great distances, which will increase antenna sizes. Tackling these two main challenges are the main focus of a great deal of the current research. For instance, in MIMO antennas, various techniques have been used to minimize mutual couplings [12,13,14,15,16,17,18,19,20,21,22,23,24,25,26,27,28,29,30]. 

Keeping the above challenges in mind, the intent of this research was to design and analyze a planar MIMO antenna, which smaller in size, with a reduced mutual coupling between the radiating elements while maintaining a high degree of performance in terms of bandwidth, gain and efficiency with a simple design structure. Considered a challenging task, mutual coupling of the radiators in MIMO communications is reduced by introducing a novel T-shaped decoupling stub over the entire bandwidth while maintaining the optimized values of ECC<0.01 and diversity gain (DG)>9.98 dB, and comparable values of gain and efficiency with a simple design structure.

## 2. Related Literature 

In rectangular-shaped MIMO antennas, isolation is increased with the help of a narrow slot that is introduced on the ground plane. The size of the antenna is 45 mm×37 mm with a $\left|S_{11}\right|$ less than −10 dB, from 3.1 to 5 GHz, which presents an impedance bandwidth of 1.9 GHz, isolation of less −20 dB, and average gain greater than −2 dB [1]. In [12], a circular-shaped MIMO antenna was studied with the overall size of 40 mm×68 mm. An inverted Y-shaped stub was used to enhance the isolation (<−15 dB). The proposed antenna resonated from 3.2 to 10.6 GHz, with an impedance bandwidth of 7.4 GHz. In [13], high isolation was achieved in a MIMO antenna with the help of an F-shaped stub. The dimensions of the design were 50 mm×30 mm, and the range of the resonance frequency was 2.5–14.5 GHz, with an impedance bandwidth of 12 GHz, an isolation less than −20 dB, ECC less than 0.04, DG greater than 7.4 dB, and multiplexing efficiency greater than −3.5 dB, respectively. In [14], a rectangular-shaped MIMO antenna with a T-shaped stub was studied to enhance isolation. The overall size of the antenna was 27 mm×47 mm. The $\left|S_{11}\right|$ was less than −10 dB in the entire UWB range, with an impedance bandwidth of 7.5 GHz. The isolation was less than −17 dB, and the maximum gain was 5.4 dBi, respectively.

A metamaterial, placed around a radiating element, can be used to improve the isolation. In [15], higher isolation was obtained with the help of a rectangular loop resonator type of metamaterial with an antenna size of 40 mm×80 mm. The impedance bandwidth of the proposed design was 4.5–8 GHz, the isolation was less than −25 dB, ECC was less than 0.02, and DG was nearly equal to 10 dB. In [16], a proposed antenna was designed for a single band of 5.8 GHz, with overall dimensions of 37 mm×44 mm. Technique using different kinds of stubs between the radiators is very helpful in obtaining significant isolation. In [17], a comb-line-shaped stub was used between U-shaped antennas to achieve a higher isolation. The dimensions of the antenna studied were 26 mm×31 mm. The antenna $\left|S_{11}\right|$ had less than −10 dB between 2.9 and 12 GHz, with an impedance bandwidth of 9.1 GHz. Another type of MIMO antenna with a T-shaped stub was used to improve isolation; the size of the studied antenna was 22 mm×31 mm. The resonance frequency of the antenna was 2.9–12 GHz and the impedance bandwidth was 9.1 GHz [18]. Similarly, mutual coupling was reduced through a defected ground structure (DGS) for a MIMO antenna operating in the frequency range of 5.77–5.96 GHz, with an impedance bandwidth of 190 MHz, isolation less than −28 dB, and ECC less than 0.003 [19].

In [20], a circular-shaped MIMO antenna was studied. A decoupling structure was introduced on both sides of the substrate. The size of the studied antenna was 4 cm×4 cm; the impedance bandwidth was 7.9 GHz, operating from 3.1 to 11 GHz. In [21], a half-circular-shaped MIMO antenna is proposed with a resonance frequency of 3.1 to 12.3 GHz. The size of the antenna was 26 mm×55 mm. In [22], mutual coupling was minimized with the help of a T-shaped stub, and the overall size of the antenna was 26 mm×28 mm×0.8 mm. In [23], two decoupling structures printed on the back of a substrate were used to obtain isolation between antennas. The antenna had a rectangular shape with a MIMO structure that presented the overall dimensions of 25 mm×38 mm, where $\left|S_{11}\right|$ was less than −10 dB, between 2.2 and 10.8 GHz. Additionally, a MIMO antenna with a circular shape was proposed for UWB applications in [24]. In that design, a single stub was used between the antennas to achieve high isolation. The overall size of the antenna was 35 mm×68 mm with a bandwidth of 7.55 GHz, operating from 3.1 to 10.65 GHz. Another type of T-shaped stub with a rectangular-shaped MIMO is proposed to reduce mutual coupling. The proposed antenna operated in the frequency range from 3.1 to 11 GHz, with a size of 22 mm×36 mm, isolation less than −15 dB, ECC less than 0.1, and where peak gain varied from 1 to 5 dBi [25]. 

In [26], the authors designed an antenna with the overall dimensions of $30\times 30\times 0.8\;{\mathrm{mm}}^{3}$ and bandwidth of 7.9 GHz. It resonated from 3.08–10.98 GHz, and isolation was achieved with the help of a stepped-shaped ground stub. The DG was equal to 9.51 dB, ECC was less than 0.013, and isolation was less or equal to −20 dB. In [27], the author proposed a rectangular-shaped fractal antenna with a T-shaped stub to reduce mutual coupling. The size of the antenna was $24\times 32\;{\mathrm{mm}}^{2}$ and the impedance bandwidth as 9.4 GHz from 3.1–12.5 GHz; the isolation wa less than −16 dB, DG was greater than 9.95 dB, and ECC was less 0.05. In [28], the authors proposed a MIMO-UWB antenna with a circular ring resonator to be used for isolation. The size was $20\times 34\times 1.6\;{\mathrm{mm}}^{3}$ and bandwidth is 8 GHz, ranging from 3–11 GHz. In [29], the authors proposed a MIMO-UWB antenna with a size of $21\times 34\times 1.6\;{\mathrm{mm}}^{3}$. A neutralization line was used between radiating patches to achieve isolation. The antenna resonated from 3.62–9.35 GHz with a bandwidth of 5.73 GHz. In [30], the author proposed a MIMO-UWB antenna with a GSM band. The size of the unit cell is$\;36\times 45\times 1.6{\;\mathrm{mm}}^{3}$. The bandwidth of the proposed UWB antenna is 9.49 GHz, and for GSM, is 0.26 GHz, respectively. The isolation is less than −20 dB, peak gain varied between 4 and 8.48 dBi, and ECC was less than 0.025. 

In [31], authors proposed a semi-circular-shaped MIMO antenna, with overall dimensions of 40×47 mm^2^, where the impedance bandwidth was 38.7 GHz, ranging from 1.3 to 40 GHz, and the isolation (<−20 dB) was increased through a T-shaped stub. The DG of the presented MIMO antenna was 10 dB, ECC was less than 0.02, and efficiency was greater than 80%. In [32], authors presented coplanar waveguide (CPW)-fed SWB-MIMO antenna with a large size of 63×63mm^2^, and the impedance bandwidth achieved is 38.7 GHz, starting from 1.3 to 40 GHz. The peak gain of the presented antenna is 5.5 dBi, the isolation is less than −16 dB, and ECC is less than 0.01. In [33], a MIMO antenna as presented with a dimension of 45×45 mm^2^, and $\left|S_{11}\right|$ was less than −10 dB in an operating range of 2.2 to 13.5 GHz, with a total impedance bandwidth of 11.3 GHz. The gain of the proposed design was 6.8 dBi, isolation was less than −18 dB, and was less than 0.01. The authors of [34] presented a UWB-MIMO antenna with a shared ground; the overall size of the antenna was 28.5×28.5 mm^2^, and a high isolation was achieved with the help of an I-shaped stub between radiators. The total impedance bandwidth of the antenna was 8.42 GHz between 2.66 and 11.08 GHz. The peak gain of the proposed MIMO antenna was 3.5 dBi, isolation was less than −15 dB, and ECC was less than 0.01. In [35], authors proposed a hexagonal-shaped MIMO antenna of a very large size (28×56 mm^2^), isolation was achieved using parasitic elements and tree distinct-shaped stubs, and the impedance bandwidth was 11.3 GHz in a range of 2 GHz to 13.3 GHz. The diversity gain was less than 9.985 dBi, ECC was less than 0.04, peak gain was 6.6 dBi, and variable radiation efficiency was between 78% to 94%. 

In [36], a rectangular-shaped MIMO antenna was discussed, where a ground stub was introduced to reduce mutual coupling, and the size of the studied antenna was 26 mm×40 mm. The impedance bandwidth was 7.5 GHz, operating from 3.1 to 10.6 GHz. The realized gain of the presented design was less than 4 dBi, ECC was less than 0.01, and isolation was less than −15 dB. In [37], a circular-fork-shaped MIMO antenna was proposed, with a resonance frequency range of 2.78 to 12.3 GHz, with a total bandwidth of 9.52 GHz, peak gain of 7 dBi, while maintaining a size of 35 mm×30 mm. In [38], rectangular radiators were placed orthogonally to minimize mutual coupling, with an overall size of antenna of 23 mm×39.8 mm, and operating frequency range of 2 to 12 GHz, with an impedance bandwidth of 10 GHz. The peak gain of the proposed design was 4 dBi, ECC was less than 0.01, and isolation was less than −20 dB. In [39], radiators and decoupling structures were printed on both sides of a substrate in order to reduce mutual coupling and the size of the antennas. The rectangular shape of the antenna with a MIMO structure presented overall dimensions of 39 mm×39 mm, and $\left|S_{11}\right|$ was less than −10 dB between 2.02 and 10.7 GHz, with a total impedance bandwidth of 8.68 GHz. The peak gains and DG of the presented antenna were 4.6 dBi and 9.5 dB, respectively, ECC was less than 0.02, isolation was less than −22 dB, and multiplexing efficiency was greater than −3 dB. Additionally, a dielectric resonator MIMO antenna with a frustum shape was proposed for SWB application by the authors of [40]. The overall size of the antenna was 95 mm×80 mm with a bandwidth of 27.6 GHz operating from 7 to 34.6 GHz. The peak gain of the presented antenna was 9.5 dBi at Port-1 and 8 dBi at Port-2; the DG was less than 9.99 dB, ECC was less than 0.01, and isolation was less than −20 dB. 

In [41], a circular-shaped MIMO antenna was proposed with overall dimensions of 50×30 mm^2^; the impedance bandwidth was 13 GHz starting from 3 to 16 GHz, the ECC was less than 0.01, peak gain was 6 dBi, and isolation was less than −16 dB. In [42], a circular-shaped MIMO antenna with a size of 64×45 mm^2^ was presented; the isolation was increased with a decoupling stub, and the impedance bandwidth was 8.5, ranging from 2.5 to 11 GHz. The gain of the proposed antenna was 6 dBi, the ECC was less than 0.02, and isolation was less than −15 dB. Various transmitters and receivers have also been designed for impulse radio UWB systems [43,44,45]. A detailed comparison between existing and proposed antennas are summarized in Table 1. 

The proposed antenna is compared with those in the literature in terms of size, bandwidth, and bandwidth dimension ratio (BDR). The proposed antenna has a very small size and a large bandwidth in comparison with the literature cited. The term BDR is a measure of what percentage of a bandwidth is provided per unit area (i.e., bandwidth range and size compactness). The BDR of the proposed antenna is 2655.86, which is better than those mentioned in the literature cited in Table 1.

## 3. Antenna Design and Characterization 

### 3.1. Antenna Design

**Figure 1:**
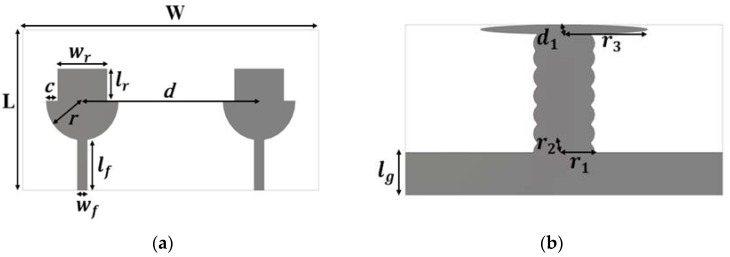
Design of MIMO antenna for SWB application. (**a**) Front view and (**b**) back view.

**Figure 2:**
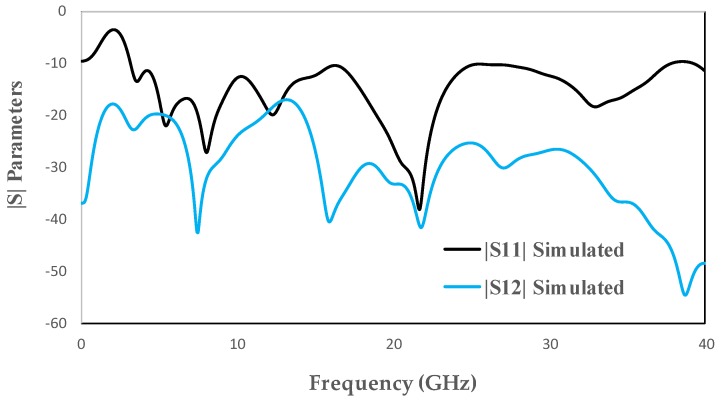
Simulated |S| parameters of MIMO antenna for SWB applications.

The top and back views of the proposed antenna are depicted in Figure 1. The |S| parameter results are depicted in Figure 2. The proposed antenna has: (i) two similar structures of a rectangular patch and a half-circular disc acting as radiating elements, (ii) a decoupling stub, and (iii) a ground plane. The overall dimensions of the proposed design are 18 mm×36 mm. The proposed design is fabricated on FR4 substrate (thickness = 1.6 mm, loss tangent = 0.02 and relative permittivity = 4.4). The radiating element consists of a rectangular patch and a half-circular disc. The radius of the half-circular disc is 4.4 mm. Similarly, the length and width of the rectangular patch are 3.6 and 6 mm, respectively. The rectangular patch is used to improve the lower cutoff frequency. The distance from one center of the radiating elements to another is 21.5 mm. The length and the width of the feed line is 5.7 and 1.2 mm, respectively. The width of the ground plane is the same as the antenna’s overall width, and the length of the ground is 4.5 mm. Various other design parameters are given in Table 2.

### 3.2. Decoupling Stub

**Figure 3:**
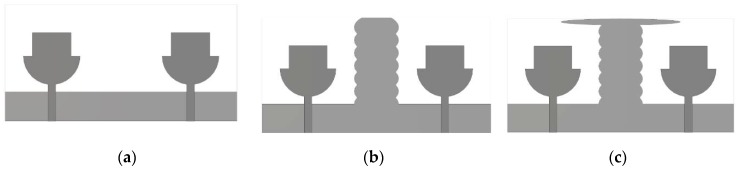
Design evaluation of decoupling stub in proposed MIMO antenna. (**a**) MIMO Ant1; (**b**) MIMO Ant2; and (**c**) MIMO Ant3.

**Figure 4:**
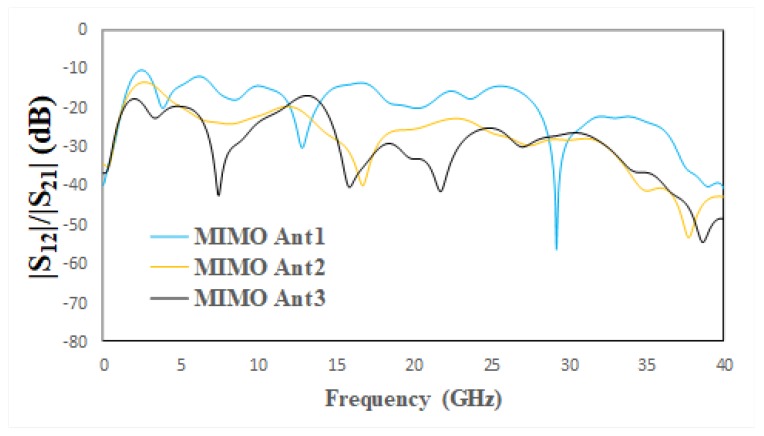
|S| Parameters of design evaluation steps of decoupling stub.

Mutual coupling between the antenna elements is a major problem of MIMO antennas that affects the diversity performance of MIMO systems. Various types of decoupling stubs are used for isolation, such as Y-shaped, F- shaped and T-shaped [6,7,8]. Hence, reducing the mutual coupling through some isolation between antennas is the main challenge in MIMO systems [46]. In this design, a decoupling stub is used for high isolation between MIMO antennas. The geometry evaluation steps of the decoupling stub are depicted in Figure 3, and the reflection coefficient of the various steps is depicted in Figure 4. In Figure 3, MIMO Ant1 has a strong mutual coupling due to the absence of a decoupling stub. For MIMO Ant2, the I-shaped decoupling stub is introduced to improve isolation between antennas due to a change in the surface current distribution. However, some mutual coupling is still observed at some frequencies, which is obvious in Figure 4. Moreover, in MIMO Ant3, an elliptical strip is added to the top of the existing stub to reduce mutual coupling on the entire frequencies, shown in Figure 4. 

**Figure 5:**
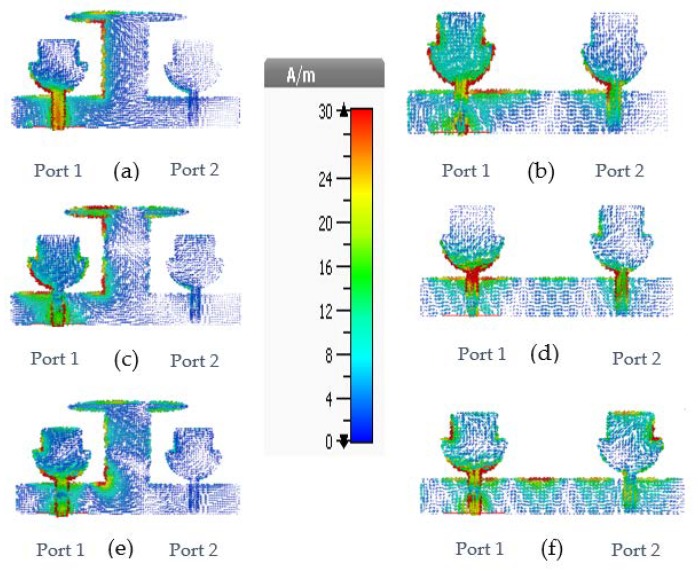
Surface current distribution of proposed MIMO antenna at (**a**) 5 GHz with decoupling stub (**b**) 5 GHz without decoupling stub (**c**) 10 GHz with decoupling stub (**d**) 10 GHz without decoupling stub (**e**) 15 GHz with decoupling stub (**f**) 15 GHz without decoupling stub.

In Figure 5, surface current distributions with and without decoupling stubs at various frequencies are also depicted. Surface current distribution is observed by exciting only port1. From Figure 5, without a decoupling stub, strong mutual coupling is observed. This is because of the coupling current proceeding from port1 to port2 in the MIMO antenna. By adding a decoupling stub, most of the current is concentrated on port1 towards the left side of the decoupling stub, which allows the interference rejection to result in high isolation on the other port. 

## 4. Results and Discussion 

**Figure 6:**
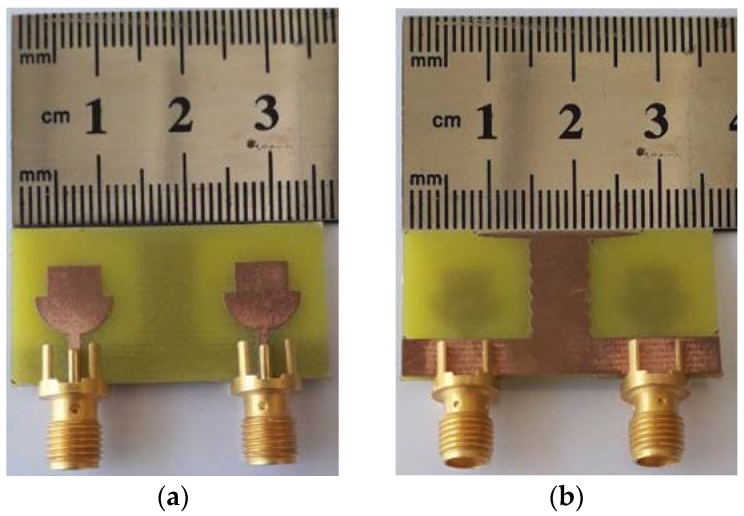
Fabricated design of proposed design. (**a**) Front view and (**b**) back view.

**Figure 7:**
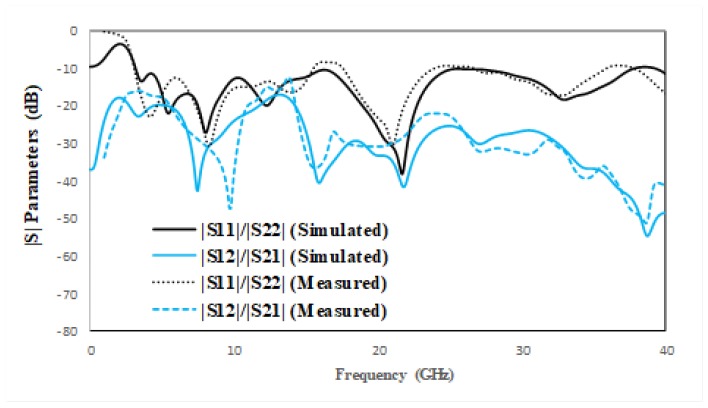
Measured and simulated |S| parameters of proposed MIMO antenna.

**Figure 8:**
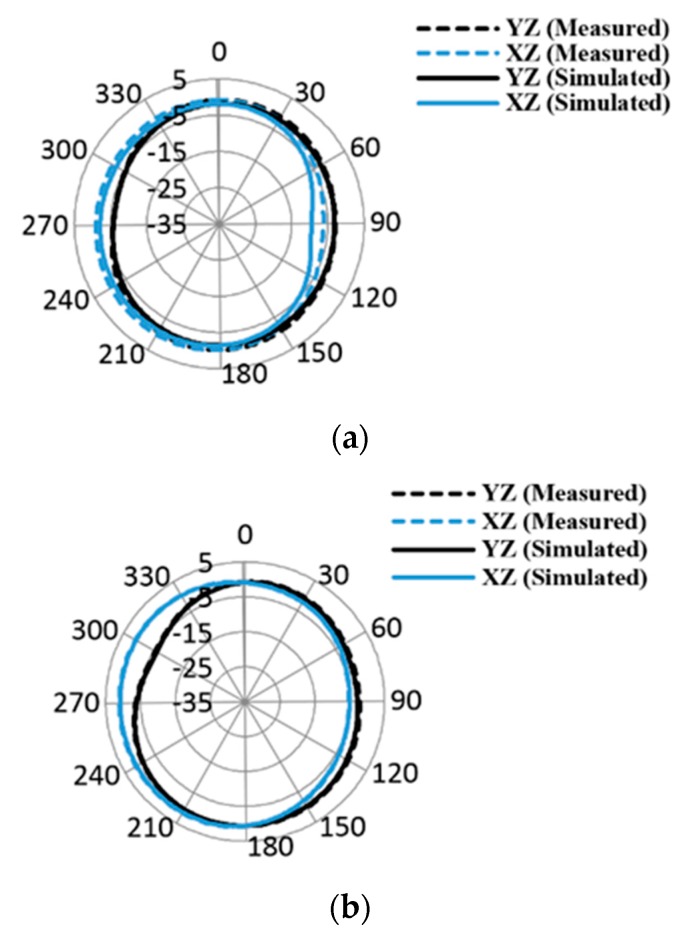

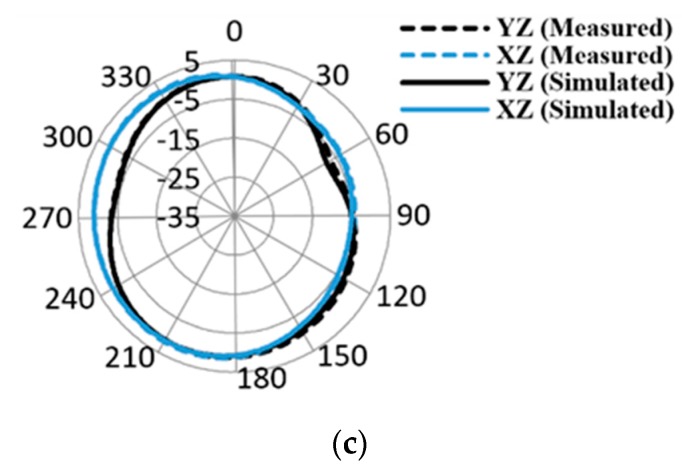
Measured and simulated radiation patterns (**a**) 3.3 GHz; (**b**) 5.5 GHz; and (**c**) 8 GHz.

The proposed antenna design has two similar structures radiating elements: a decoupling stub and a ground plane. The proposed design is simulated and optimized in CST Microwave Studio (CST-MWS), an electromagnetic computer simulation program. The front and back view of fabricated MIMO antenna is depicted in Figure 6. The measured and simulated |S| parameters are compared in Figure 7. It shows significant agreement between simulated and measured results. The antenna is radiating between 3 and 40 GHz. The simulated and measured radiation pattern at various frequencies in both E Plane (YZ, *ϕ* = 90) and H Plane (XZ, *ϕ* = 0) are compared in Figure 8. The radiation pattern at 3.5 GHz is nearly omni-directional in both the YZ Plane and XZ Plane, the radiation patterns at 5.5 and 8 GHz also form an omni directional pattern in the YZ Plane and XZ Plane. The radiation patterns are stable at the given frequencies, which is justified in Figure 8. 

Diversity gain (DG) and ECC are the most important performance parameters for the capability of a MIMO-UWB antenna. A value of ECC is ideally equal to zero, and, practically, ECC < 0.5 for an uncorrelated MIMO antenna. ECC and DG are calculated from the following formula [47].

(1) $$ECC=\frac{{\left|S_{11}^{*}S_{12}+S_{21}^{*}S_{22}\right|}^{2}}{\left(1-{\left|S_{11}\right|}^{2}-{\left|S_{21}\right|}^{2}\right)\left(1-{\left|S_{22}\right|}^{2}-{\left|S_{12}\right|}^{2}\right)}$$

(2) $$\mathrm{DG}=10\sqrt{1-{\left(ECC\right)}^{2}}$$

**Figure 9:**
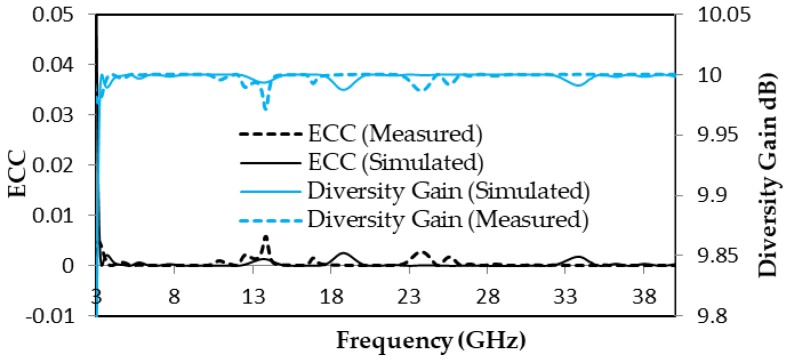
ECC and diversity gain of proposed antenna.

**Figure 10:**
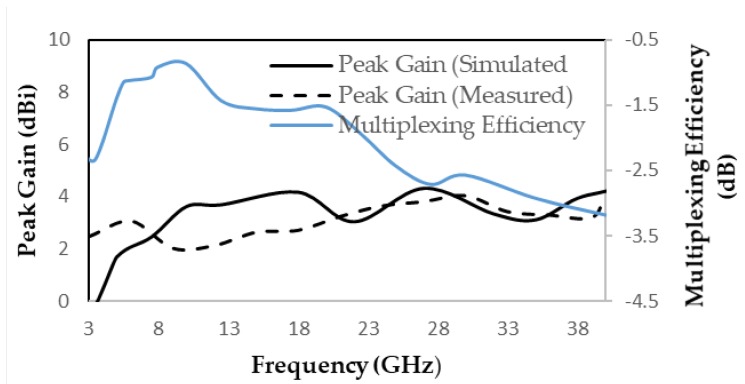
Multiplexing efficiency and peak gain of proposed MIMO antenna.

The measured and simulated ECC is < 0.01 of the proposed antenna, which can be justified from Figure 9. The measured and simulated diversity gain is seen in Figure 9 that DG > 9.98 dB. The peak gain and multiplexing efficiency are given in Figure 10, where measured and simulated peak gain varies between 0 and 4 dBi over the entire frequencies. The multiplexing efficiency of the proposed design varies between −1 and −3.5 dB over entire frequencies, which is justified from Figure 10.

## 5. Conclusions

In this work, a new and more compact MIMO antenna with a novel-structured decoupling stub, enhanced bandwidth (37 GHz), and high isolation is presented. The size of the antenna is as small as 18 mm×36 mm, while the isolation is increased by introducing a decoupling stub. The peak gain varies between 0 and 9 dB and achieved a comprehensive agreement between theoretical and measured results. The multiplexing efficiency, peak gain, ECC, and DG show that the proposed antenna can be used for various MIMO-UWB wireless applications.

## Figures and Tables

**Table 1:** Comparison between previously presented MIMO antennas with proposed antennas.

Ref.	Year	Size (mm × mm)	Resonance Freq: Range (GHz)	BW:1	% BW	BDR
[1]	2009	45×37	3.1–5	1.61	47	264.36
[12]	2011	40×68	3.2–10.6	3.31	107.24	346.52
[13]	2018	50×30	2.5–14.5	5.8	141.17	1355.23
[14]	2013	27×47	3.1–10.6	3.41	109.50	808.11
[15]	2019	40×80	4.5–8	1.78	56	77.77
[17]	2016	26×31	2.9–12	4.13	122.14	1621.69
[18]	2018	22×31	2.9–12	4.13	122.14	1916.55
[20]	2017	40×40	3.1–11	3.54	112	655.56
[21]	2015	26×55	3.1–12.3	3.96	119.5	782.62
[22]	2019	26×28	2.9–10.8	3.72	115.32	1695.19
[23]	2018	25×38	2.2–10.8	4.8	141	2759.89
[24]	2016	35×68	3.1–10.65	3.43	109.89	432.41
[25]	2015	22×36	3.1–11	3.54	112	1324.37
[26]	2017	30×30	3.08–10.98	3.56	112.37	1184.53
[27]	2019	24×32	3.1–12.5	4.03	120.51	1469.53
[28]	2019	20×34	3.1–11	3.54	112	1542.51
[29]	2019	21×34	3.62–9.35	2.58	88.35	849.83
[30]	2019	36×45	3.01–12.5	4.15	122.37	750.35
Proposed	2020	18×36	3–40	13.33	172.1	2655.86

**Table 2:** Parameters of proposed MIMO-UWB antenna.

Parameter	Value (mm)	Parameter	Value (mm)
W	36	r	4.4
L	18	$r_{1}$	3.5
$l_{f}$	5.7	$r_{2}$	2
$l_{r}$	3.6	$r_{3}$	9.5
$l_{g}$	4.5	d	21.5
$w_{f}$	1.2	$d_{1}$	1
$w_{r}$	6	C	1.4
